# Co-Circulation of Different Hepatitis E Virus Genotype 3 Subtypes in Pigs and Wild Boar in North-East Germany, 2019

**DOI:** 10.3390/pathogens11070773

**Published:** 2022-07-06

**Authors:** Grit Priemer, Filip Cierniak, Carola Wolf, Rainer G. Ulrich, Martin H. Groschup, Martin Eiden

**Affiliations:** 1 Department 2—Animal Disease Diagnostics, State Office for Agriculture, Food Safety and Fisheries Mecklenburg—Western Pomerania, 18059 Rostock, Germany; grit.priemer@lallf.mvnet.de (G.P.); carola.wolf@lallf.mvnet.de (C.W.); 2 Institute of Novel and Emerging Infectious Diseases at the Friedrich-Loeffler-Institut, Federal Research Institute for Animal Health, 17493 Greifswald-Insel Riems, Germany; filip.cierniak@outlook.de (F.C.); rainer.ulrich@fli.de (R.G.U.); martin.groschup@fli.de (M.H.G.); 3 Partner Site Hamburg-Lübeck-Borstel-Riems, German Centre for Infection Research (DZIF), 17493 Greifswald-Insel Riems, Germany

**Keywords:** *Hepeviridae*, genotype, HEV-3, subtype, reservoir, transmission, One Health

## Abstract

Hepatitis E is a major cause of acute liver disease in humans worldwide. The infection is caused by hepatitis E virus (HEV) which is transmitted in Europe to humans primarily through zoonotic foodborne transmission from domestic pigs, wild boar, rabbits, and deer. HEV belongs to the family *Hepeviridae*, and possesses a positive-sense, single stranded RNA genome. This agent usually causes an acute self-limited infection in humans, but in people with low immunity, e.g., immunosuppressive therapy or underlying liver diseases, the infection can evolve to chronicity and is able to induce a variety of extrahepatic manifestations. Pig and wild boar have been identified as the primary animal reservoir in Europe, and consumption of raw and undercooked pork is known to pose a potential risk of foodborne HEV infection. In this study, we analysed pig and wild boar liver, faeces, and muscle samples collected in 2019 in Mecklenburg-Western Pomerania, north-east Germany. A total of 393 animals of both species were investigated using quantitative real-time reverse transcription polymerase chain reaction (RT-qPCR), conventional nested RT-PCR and sequence analysis of amplification products. In 33 animals, HEV RNA was detected in liver and/or faeces. In one individual, viral RNA was detected in muscle tissue. Sequence analysis of a partial open reading frame 1 region demonstrated a broad variety of genotype 3 (HEV-3) subtypes. In conclusion, the study demonstrates a high, but varying prevalence of HEV RNA in swine populations in Mecklenburg-Western Pomerania. The associated risk of foodborne HEV infection needs the establishment of sustainable surveillance and treatment strategies at the interface between humans, animals, and the environment within a One Health framework.

## 1. Introduction

Hepatitis E virus (HEV) belongs to the *Hepeviridae* family and is the most common cause of acute viral hepatitis throughout the world [1]. In total, 20 million HEV infections occur each year, with over three million acute cases and 44,000 hepatitis E-related deaths [2]. Hepeviruses are subdivided into the subfamilies *Orthohepevirinae* and *Parahepevirinae* with fish-infecting hepeviruses. Most human pathogenic genotypes are grouped within the species *Orthohepevirus balayani* [3]. These are the exclusively human-associated genotypes HEV-1 and HEV-2 as well as the zoonotic genotypes HEV-3/HEV-4, which circulate between animal reservoirs and humans. In contrast to HEV-3, which is endemic in Europe, there are only few reports of HEV-4 in Europe so far. This includes infection of pigs in Belgium [4] and human cases in Germany in 2007 [5], Italy in 2011 [6] and France in 2014 [7]. Recently, the camel-associated genotype HEV-7 has been detected in a human patient [8].

Genera *Avihepevirus* comprises avian, and *Chirohepevirus* bat-associated hepevirus strains. Genus *Rocahepevirus* comprises rodent and mustelid borne hepeviruses; rat HEV (species *Orthohepevirus ratti*)-related infections of patients have been described recently [9,10,11], demonstrating the zoonotic potential of this hepevirus.

HEV is highly endemic in regions in Africa, Asia, or the Middle East and causes large epidemics of acute hepatitis in these regions, mainly due to poor sanitation and contaminated drinking water. One major route of transmission in developed countries is zoonotic [12]. In Europe, which is almost exclusively dominated by genotype HEV-3, the consumption of contaminated and undercooked food, especially pork and meat products, is the most common cause of infection [13]. Transfer of blood, blood products, and organ transplantations are additional routes of transmission and infection [14].

The HEV genome is a single-stranded positive-sense RNA genome of approximately 7.2 kb. The open reading frame 1 (ORF1) at the 5’ end of the genome encodes the nonstructural polyprotein. ORF2 encodes the capsid protein and is located at the 3′ end of the viral genome. ORF3 encodes a small multifunctional protein and overlaps with the 5′ end of ORF2 [15]. This overlapping region is highly conserved and can be used for molecular detection of HEV RNA [16,17]. Additionally, the 5′ end of the viral genome acts as binding site for the viral RNA-directed RNA polymerase (RdRP), is also highly conserved, and can therefore be used as a target for molecular detection as well [17].

HEV-3 infections of humans through ingestion of contaminated, undercooked animal products have been thoroughly investigated and provide evidence for a broad spectrum of animal species including swine, deer, rabbit, and camel as source of infection [13]. In Europe, this mainly comprises wild boars and pigs, but also rabbits and deer [18]. In Germany, rabbits show high detection rates with a rabbit-specific subtype (HEV-3ra) displaying a RNA prevalence of 17 to 25% [19,20,21]. Isolated human cases of infection, especially in France with this genotype, demonstrate the zoonotic potential of this virus variant [22]. In contrast, the German roe and red deer populations exhibit only low detection rates of both HEV-specific antibodies and RNA [23], which suggests a rather minor importance of these animal species as virus reservoirs for human infections in Germany so far.

In our study, liver and muscle tissue samples and faeces from 318 pigs and 75 wild boars were collected during 2019 by the State Office for Agriculture, Food Safety and Fisheries Mecklenburg-Western Pomerania (Landesamt für Landwirtschaft, Lebensmittelsicherheit und Fischerei Mecklenburg-Vorpommern, LALLF) and provided for HEV diagnostic investigations. Viral RNA was detected in 27 farmed pigs and 6 wild boars, including one muscle sample, which demonstrates a continuing high prevalence of HEV in farmed pigs and wild boar in the north-east of Germany.

## 2. Materials and Methods

### 2.1. Collection of Wild Boar and Pig Samples

Wild boar and pig liver and muscle samples were collected over the entire year 2019 by the LALLF and monthly shipped to FLI for further analysis. Muscle tissue originates from lateral femoral musculature (musculus biceps femoris). Accompanying faeces samples were collected between August and December 2019 (see Supplementary Table S1).

### 2.2. RNA Isolation

RNA extraction from liver and muscle tissue samples was performed with the Qiagen RNeasy Kit (Qiagen, Hilden, Germany) according to manufacturer’s instructions. Liver and muscle tissues were freshly prepared during dissection and immediately frozen. For RNA extraction, MS2 bacteriophages were added to the tissue and faeces samples. RNA was then isolated with the RNeasy kit using guanidine thiocyanate and selective binding of RNA on a silica-based membrane, which removes most of the potential inhibitors, enables efficient RNA recovery and is especially recommended for liver and muscle tissues. From faecal samples, a 10% suspension was made with 0.89% NaCl-solution. After vortexing and centrifugation (4400× *g*, 4 °C, 20 min), the supernatant was sterile filtrated using a sterile 0.22 μm MILLEX-GP Syringe Filter Unit (Millipore, Tullagreen, Ireland) and subjected to RNA isolation using the QIAamp viral RNA Kit (Qiagen, Hilden, Germany).

### 2.3. Quantitative Real-Time RT-PCR and Sequence Analysis

HEV RNA was detected with a quantitative real-time RT-PCR (RT-qPCR), which targets a sequence within a conserved overlapping ORF2/ORF3 region, and determined by cycle threshold (ct) values [17]. The following primers and probes were used: forward primer (5′-GTGCCGGCGGTGGTTTCTG-3′), reverse primer (5′-GCGAAGGGGTTGGTTGGATG -3′) and probe 5′-FAM-TGACMGGGTTGATTCTCAGCC-BHQ1-3′). As internal RNA extraction and RT-qPCR control, RNA bacteriophage MS2 particles were added to each sample to exclude false negative results in accordance with [24]. MS2 bacteriophage derived RNA was detected using primers MS2F (5′-CTCTGAGAGCGGCTCTATTGGT-3′), MS2R (5′-GTTCCCTACAACGAGCCTAAATTC-3′) and MS2 probe (5′-HEX-TCAGACACGCGGTCCGCTATAACGA-BHQ1-3′). The assays were carried out following the Minimum Information for Publication of Quantitative Real-Time PCR Experiments (MIQE) guidelines [25]. For phylogenetic analysis, partial sequences were amplified targeting an ORF1 region (nucleotide positions 127–376; numbering refers to FJ705359) using a nested RT-PCR protocol with first round primers HEV.ORF1_F1 (5′-CCCAYCAGTTYATWAAGGCTCCTGGC-3′) and HEV.ORF1_R1 (5′-TGCARDGARTANARRGCNAYNCCNGTCTC-3′) followed by second round primers HEV.ORF1_F2 (5′-AAYTCYGCCYTGGCGAATGCTGTGGTGGT-3′) and HEV.ORF1_R2 (5′-CCVCGRGTNG GRGCRGWRTACCA-3′). In brief, reverse transcription was carried out with Superscript^®^ III Reverse Transcriptase (Thermo Fisher Scientific Inc., Waltham, MA, USA) and the subsequent nested PCR with Maxima SYBR Green/Fluorescein qPCR Master Mix Kit (Thermo Fisher Scientific Inc., USA). Finally, a melting curve analysis was performed starting with a temperature gradient from 68 to 94 °C in steps of 0.2 °C. Positive samples were identified by melting peaks and amplicons were subsequently sequenced (Eurofins Genomics, Munich, Germany). Detailed protocol is found in a previous publication [17].

### 2.4. Phylogenetic Analysis

Reference sequences for HEV-3 subtypes were selected according to [26]. The sequences were aligned using, with Minimap2 [27], using HEV-1 (GenBank acc. no. M73218) as reference (full HEV-3 sequence set). Multiple alignments of amplicon sequences were made using MUSCLE in MEGA 11 [28,29] and subsequently manually inserted into the reference alignment using Ugene [30]. The phylogenetic analysis of the amplicon region was performed with MEGA 11 using the Maximum Likelihood method and General Time Reversible (GTR) model. A discrete gamma distribution was used to model evolutionary rate differences among sites (5 categories). The rate variation model allowed for some sites to be evolutionarily invariable. Tree visualisation was done in MEGA and in R [31] using Rstudio [32] with packages dplyr [33], ggplot2 [34], ggtree [35,36,37], and treeio [38].

## 3. Results

In 2019, samples from 317 pigs and 76 wild boars were collected by routine sampling in the framework of a disease monitoring program by the LALLF and transferred to FLI. Liver, muscle, and faecal samples were submitted from 179 animals, liver, and muscle tissue from 212 animals, from one individual-only faeces, and from one individual-only muscle tissue. A compilation of collected samples is displayed in Supplementary Table S1. All samples were subjected to RNA isolation followed by HEV-specific RT-qPCR. To avoid false negative results due to RT-PCR inhibitors within the tissue or faeces samples a silica bead-based RNA extraction method of freshly prepared samples was performed to which a bacteriophage MS2 based RNA extraction control was added. A multiplex RT-qPCR was performed detecting HEV RNA, in the FAM channel and the MS2-RNA in the HEX channel, which provides the validity of the RT-PCR reaction.

**Figure 1:**
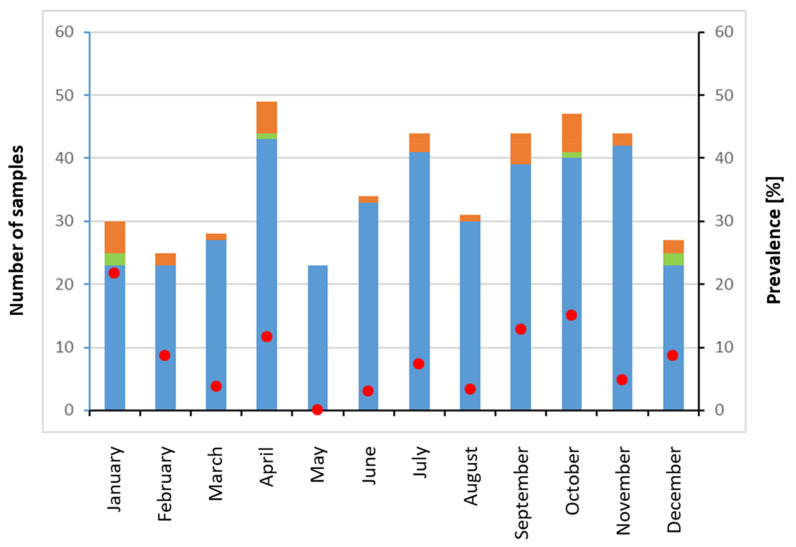
Number of pigs and wild boars collected per month (blue and green column, respectively) with the respective number of HEV RNA-positive liver (orange bar) and corresponding prevalence rates (red dots). Animals were sampled in 2019 in Mecklenburg-Western Pomerania, Germany.

**Figure 2:**
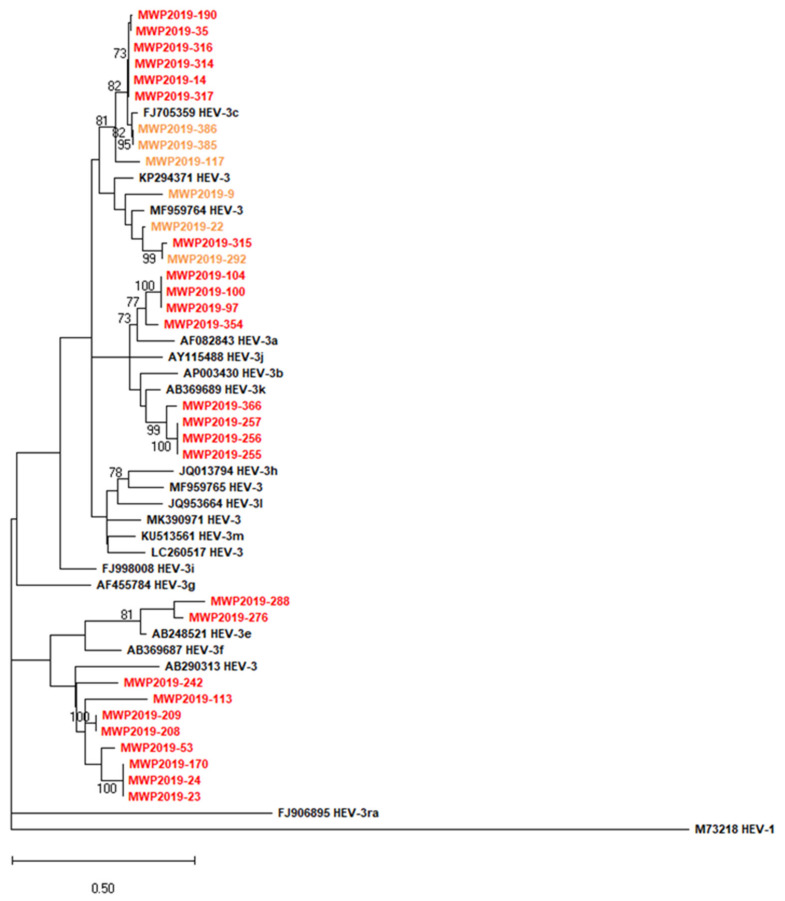
Phylogenetic relationship of HEV sequences from domestic pig and wild boar livers or faeces (MWP2019-385). The phylogenetic tree is based on the 250 nucleotide ORF1 region of HEV (nucleotide positions 127–376 of reference sequence FJ705359). The tree is drawn to scale, as the evolutionary distances used to derive the phylogenetic tree. The sequences retrieved from the NCBI GenBank are given with accession numbers. The HEV sequences obtained in this study from pigs are in red, sequences obtained from wild boar are in orange. Reconstruction of phylogenetic tree using Maximum Likelihood method with 500 bootstrap iterations. Bootstrap values >70 are annotated.

In total, in 33 individuals,27 farmed pigs and 6 wild boars, viral RNA was detected either in liver (*n* = 31) and/or accompanying faeces (*n* = 14) (Table 1). The monthly number of analysed individuals varied between 23 and 44. The number of HEV RNA-positive animals varied between 0 and 6, resulting in monthly prevalences of 0 to 14.6% (mean 8.14%; 95% confidence interval, CI, 5.66–10.62%) over the year (Figure 1). In one case, in addition to liver, viral RNA could be detected in a muscle sample exhibiting a low ct value of 20.1. RT-PCR detection of HEV RNA followed a nested SYBR Green RT-PCR protocol followed by sequencing of generated amplicons at Eurofins (Munich, Germany). For 31 out of 33 animals, a partial sequence from the ORF1 region of the genome could be recovered which was used for phylogenetic analysis with MEGA 11 (Figure 2).

The examination revealed for each sequence an affiliation to HEV-3 genotype, but a high variety of HEV-3 subtypes. In total, six different subtypes were identified in pig and wild boar including subtypes 3a (*n* = 4 pigs/0 wild boar), 3c (*n* = 6 pigs/3 wild boars), 3e (*n* = 2 pigs/0 wild boar), 3f (*n* = 8 pigs/0 wild boar), and 3k (*n* = 4 pigs/0 wild boar).

One set of four (1 pig/3 wild boars) sequences (3i-like) cluster with wild boar strains (MF959764, KP294371) that have not been assigned to a subtype by Smith et al. [26], but were assigned to subtype 3i in a more detailed analysis of HEV-3 [39]. A detailed phylogenetic tree based on recovered partial sequences is deposited as Supplementary Figure S1. In general, when both liver and faecal samples were HEV RNA-positive, the ct value (as proxy for viral load) was approximately the same (*n* = 11). The two exceptions iare pig MWP2019-255 with ct values of 31.34 (liver) vs. 38.2 (faeces).and pig MWP2019-257 with ct values of 36.65 (liver) vs. 40.71 (faeces).

## 4. Discussion

In total, 393 animals were investigated for HEV RNA, yielding a prevalence of 8.5% in domestic pigs and 7.9% in wild boars. The results confirmed the circulation of HEV-3 in pigs and wild boar in the federal state of Mecklenburg-Western Pomerania located in north-east Germany on the Baltic Sea coast. The results are in line with previous findings in the same region from 1996/1997 and 2005/2006 where viral RNA was detected in wild boar blood samples at prevalences of approximately 3.4% and 5.2%, and in addition, in wild boar livers from the Greifswald region with a prevalence of 10.4%, respectively [17].

Several studies in Germany assigned domestic pigs and wild boars as main HEV reservoirs, which is reflected by high seroprevalence rates in farmed pigs, ranging from 42.7% and 49.8%, up to 100% [40,41,42] and 33%, up to 41% in wild boars [43,44]. High rates in pigs were also found throughout Europe with seroprevalences from 20.4% in Spain [45], 45.1% in Italy [46], 60% in France [47], 70% in The Netherlands [48], and 92.8% in the United Kingdom [49]. These high prevalences indicate the significant risk for developing HEV infection after consumption of pork. This route of transmission has been directly confirmed in case and outbreak investigations, in which the same virus strain was detected both in the patient and in the consumed food. In France, an outbreak of hepatitis E was described in seven people infected by eating traditional sausage (“Figatellu”) containing raw pork portions [50]. A similar case has also been reported in Switzerland, which occurred after consumption of a different type of sausage containing raw pork liver [51]. Another report also confirmed the presence of infectious virus in pork liver sausage from southern France [52]. For Germany, several studies have been conducted that identified the consumption of offal and wild boar meat [5], ready-to-eat pork products [53] and sausages [54] as the highest risk factor for hepatitis E disease. Especially liver and liver sausages are main infectious food sources, which is reflected by high viral RNA prevalences ranging from 5.2% in blood [17], over 18% in liver up to 56.3% in bile samples of wild boars [55] as well as 13.5% in pig derived liver samples [56] and 22.0% in liver sausages from Germany [57]. Similar observations have been made throughout Europe [13].

HEV strains from 31 out of 33 HEV positive animals could be genotyped and ex-hibited a great variety of HEV-3 subtypes including 3a, 3c, 3e, 3f, and 3k. Most of the subtypes (3a, 3c, 3e, 3f) have been regularly found in pigs from Germany and were also detected in humans in Germany [17], which indicates that pigs are a probable source of human infections. Regarding subtype 3k, there is only one notification in Europe from pigs from Slovenia [17] and otherwise reports from human strains in Japan [58]. Additionally, sequences of four samples, designated as HEV-3i-like, cluster with MF959764 and KP294371, sequences which were not assigned to a subtype proposed by Smith et al. [26]. However, a more recent analysis groups both sequences with subtype HEV-3i [39], Notably, KP294371 was detected in a wild boar in Mecklenburg-Western Pomerania in 2010 [59,60]. In each case, the faecal samples that accompanied the positive liver samples were also positive and recovered sequences were in virtually all cases highly similar or identical (Figure S1). For further conclusions on the transmission and circulation of HEV strains between humans, pigs, and wild boars in Germany, a higher number of German porcine-derived HEV sequences would be supportive.

The detection of HEV RNA with high load in a muscle tissue from one pig underlines the public health risks associated with HEV as a foodborne pathogen. The analysed muscle derives from lateral femoral musculature, which is often supplied as high-quality meat, e.g., steaks. So far, HEV RNA presence in muscle of naturally infected pig was documented only in few studies: In one study from Spain, a HEV-positive diaphragm in 1 out of 225 slaughterhouse pigs was detected [61] and a second surveillance documented 1 and 2 positive lingual muscle samples collected in slaughterhouses from Czech Republic and Italy, respectively [62]. A second report from Italy detected 8 positive diaphragm muscles from 585 slaughtered pigs [63]. A positive diaphragm muscle (1/45) was also detected in slaughtered pigs from Spain [64]. No viral RNA could be observed in muscle tissue from slaughterhouse samples in Canada (Leblanc et al. [65]; number of samples: *n* = 43), France ([47], *n* = 1134) and Denmark ([66]; *n* = 10). In contrast, multiple findings were notified in muscle tissue samples of German wild boar and deer [67] and wild boar muscle tissues from Italy [68]. However, in such studies, attention must be given to the risk of cross-contamination of carcass surfaces during the dissection as notified by others [69,70]. In our study, however, the muscle sample had a very low ct value compared to the liver (ct value 20.7 vs. 23.1) and was prepared during dissection by an experienced pathologist for subsequent examination, which minimised the risk of contamination. The detected HEV strain in pig muscle belongs to subtype 3f which needs further attention because this subtype has been assigned to acute human HEV cases in France [71] and higher risk of hospitalisation in Belgium [72] as well as was involved in a hepatitis E outbreak in Italy 2019 [73].

In conclusion, this study highlights the need for the implementation of control measures including continuous surveillance and monitoring of HEV in domestic pigs and wild boars throughout Germany. Within the framework of a One Health concept, further epidemiology studies should consider the inclusion of human specimen and elucidate the interplay of HEV subtypes on acute hepatitis, hospitalisation, and chronic courses of HEV infections.

## Figures and Tables

**Table 1:** Summary of positive animals, including ct values from liver, faeces, and muscle samples as well as subtype annotation of the HEV sequence.

No.	Sample ID	Species	Tissue (ct-Value)			Genotype	Subtype	Accession
			Liver	Faeces	Muscle			Number
1	MWP2019-9	wb	32.7	n.a.	neg	HEV-3	3i-like	ON240936
2	MWP2019-14	pig	21.3	n.a.	neg	HEV-3	3c	ON240935
3	MWP2019-22	wb	22.86	n.a.	neg	HEV-3	3i-like	ON240934
4	MWP2019-23	pig	26.52	n.a.	neg	HEV-3	3f	ON240933
5	MWP2019-24	pig	24.14	n.a.	neg	HEV-3	3f	ON240932
6	MWP2019-33	pig	32.36	n.a.	neg	no sequence	-	-
7	MWP2019-35	pig	19.23	n.a.	neg	HEV-3	3c	ON240931
8	MWP2019-53	pig	27	n.a.	neg	HEV-3	3f	ON240930
9	MWP2019-97	pig	26.2	n.a.	neg	HEV-3	3a	ON240929
10	MWP2019-100	pig	22.2	n.a.	neg	HEV-3	3a	ON240928
11	MWP2019-104	pig	33.1	n.a.	neg	HEV-3	3a	ON240927
12	MWP2019-113	pig	32.8	n.a.	neg	HEV-3	3f	ON240926
13	MWP2019-117	wb	28.1	n.a.	neg	HEV-3	3c	ON240925
14	MWP2019-170	pig	23.1	n.a.	20.7	HEV-3	3f	ON240924
15	MWP2019-190	pig	27.4	n.a.	neg	HEV-3	3c	ON240923
16	MWP2019-208	pig	23.5	n.a.	neg	HEV-3	3f	ON240922
17	MWP2019-209	pig	32.4	n.a.	neg	HEV-3	3f	ON240921
18	MWP2019-242	pig	30.4	29.19	neg	HEV-3	3f	ON240949
19	MWP2019-255	pig	31.34	38.20	neg	HEV-3	3k	ON240948
20	MWP2019-256	pig	34.62	34.19	neg	HEV-3	3k	ON240947
21	MWP2019-257	pig	36.65	40.71	neg	HEV-3	3k	ON240946
22	MWP2019-276	pig	21.26	20.26	neg	HEV-3	3e	ON240945
23	MWP2019-277	pig	n.a.	34.68	neg	no sequence	-	-
24	MWP2019-288	pig	22.86	24.56	neg	HEV-3	3e	ON240943
25	MWP2019-292	wb	23.95	27	neg	HEV-3	3i-like	ON240942
26	MWP2019-314	pig	22.8	22.69	neg	HEV-3	3c	ON240950
27	MWP2019-315	pig	22.1	22.39	neg	HEV-3	3i-like	ON240941
28	MWP2019-316	pig	23.29	21.19	neg	HEV-3	3c	ON240940
29	MWP2019-317	pig	20.18	22.1	neg	HEV-3	3c	ON240939
30	MWP2019-354	pig	23.56	24.19	neg	HEV-3	3a	ON240938
31	MWP2019-366	pig	28.05	n.a	neg	HEV-3	3k	ON240937
32	MWP2019-385	wb	n.a.	23.51	neg	HEV-3	3c	ON240944
33	MWP2019-386	wb	16.3	n.a.	neg	HEV-3	3c	ON240944

wb, wild boar; n.a., no sample available; neg, negative.

## Data Availability

All relevant information is contained within the manuscript or the Supplementary Materials. The novel RNA sequences were uploaded at GenBank with the accession numbers ON240921, ON240922, ON240923, ON240924, ON240925, ON240926, ON240927, ON240928, ON240929, ON240930, ON240931, ON240932, ON240933, ON240934, ON240935, ON240936, ON240937, ON240938, ON240939, ON240940, ON240941, ON240942, ON240943, ON240944, ON240944, ON240945, ON240946, ON240947, ON240948, ON240949, and ON240950.

## Supplementary Materials

The following supporting information can be downloaded at: https://www.mdpi.com/article/10.3390/pathogens11070773/s1, Figure S1: Molecular phylogenetic analysis of 139 250-nucleotide-long partial ORF1 HEV-3 sequences corresponding to the region 127–376 of reference sequence FJ705359. Table S1: Summary of the data for all sampled domestic pig and wild boar.
