# Supplementary material for: Co-Circulation of Different Hepatitis E Virus Genotype 3 Subtypes in Pigs and Wild Boar in North-East Germany, 2019
Source: Pathogens. 2022 Jul 6;11(7):773. doi: 10.3390/pathogens11070773 (PMC9317891; doi:10.3390/pathogens11070773)
Supplement: Supplementary file 1 [file pathogens-11-00773-s001.zip › pathogens-1773450-supplementary.pdf]

Table S1. Summary of the data for all sampled domestic pig and wild boar.

numbering	date of collection	species	liver	feces	muscle
MWP2019-1	02.01.2019	pig	X		X
MWP2019-2	02.01.2019	pig	X		X
MWP2019-3	07.01.2019	pig	X		X
MWP2019-4	07.01.2019	pig	X		X
MWP2019-5	07.01.2019	pig	X		X
MWP2019-6	08.01.2019	pig	X		X
MWP2019-7	09.01.2019	pig	X		X
MWP2019-8	09.01.2019	pig	X		X
MWP2019-9	11.01.2019	wild boar	X		X
MWP2019-10	14.01.2019	pig	X		X
MWP2019-11	14.01.2019	pig	X		X
MWP2019-12	14.01.2019	pig	X		X
MWP2019-13	15.01.2019	pig	X		X
MWP2019-14	15.01.2019	pig	X		X
MWP2019-15	17.01.2019	pig	X		X
MWP2019-16	17.01.2019	pig	X		X
MWP2019-17	17.01.2019	pig	X		X
MWP2019-18	22.01.2019	pig	X		X
MWP2019-19	22.01.2019	pig	X		X
MWP2019-20	22.01.2019	wild boar	X		X
MWP2019-21	22.01.2019	wild boar	X		X
MWP2019-22	24.01.2019	wild boar	X		X
MWP2019-23	30.01.2019	pig	X		X
MWP2019-24	30.01.2019	pig	X		X
MWP2019-25	31.01.2019	pig	X		X
MWP2019-26	05.02.2019	pig	X		X
MWP2019-27	06.02.2019	pig	X		X
MWP2019-28	07.02.2019	pig	X		X
MWP2019-29	08.02.2019	pig	X		X
MWP2019-30	08.02.2019	pig	X		X
MWP2019-31	12.02.2019	pig	X		X
MWP2019-32	12.02.2019	pig	X		X
MWP2019-33	12.02.2019	pig	X		X
MWP2019-34	12.02.2019	pig	X		X
MWP2019-35	12.02.2019	pig	X		X
MWP2019-36	18.02.2019	pig	X		X
MWP2019-37	18.02.2019	pig	X		X
MWP2019-38	19.02.2019	pig	X		X
MWP2019-39	19.02.2019	pig	X		X
MWP2019-40	20.02.2019	pig	X		X
MWP2019-41	22.02.2019	pig	X		X
MWP2019-42	25.02.2019	wild boar	X		X
MWP2019-43	27.02.2019	pig	X		X
MWP2019-44	28.02.2019	pig	X		X
MWP2019-45	27.02.2019	pig	X		X
MWP2019-46	27.02.2019	pig	X		X
MWP2019-47	27.02.2019	pig	X		X
MWP2019-48	28.02.2019	pig	X		X
MWP2019-49	01.03.2019	pig	X		X
MWP2019-50	01.03.2019	pig	X		X
MWP2019-51	01.03.2019	pig	X		X

MWP2019-52	04.03.2019	pig	X		X
MWP2019-53	05.03.2019	pig	X		X
MWP2019-54	05.03.2019	pig	X		X
MWP2019-55	06.03.2019	pig	X		X
MWP2019-56	06.03.2019	pig	X		X
MWP2019-57	06.03.2019	pig	X		X
MWP2019-58	06.03.2019	pig	X		X
MWP2019-59	06.03.2019	pig	X		X
MWP2019-60	07.03.2019	wild boar	X		X
MWP2019-61	08.03.2019	pig	X		X
MWP2019-62	08.03.2019	pig	X		X
MWP2019-63	12.03.2019	wild boar	X		X
MWP2019-64	12.03.2019	pig	X		X
MWP2019-65	12.03.2019	pig	X		X
MWP2019-66	21.03.2019	pig	X		X
MWP2019-67	21.03.2019	pig	X		X
MWP2019-68	21.03.2019	pig	X		X
MWP2019-69	21.03.2019	wild boar	X		X
MWP2019-70	25.03.2019	pig	X		X
MWP2019-71	25.03.2019	pig	X		X
MWP2019-72	25.03.2019	pig	X		X
MWP2019-73	25.03.2019	pig	X		X
MWP2019-74	26.03.2019	pig	X		X
MWP2019-75	26.03.2019	pig	X		X
MWP2019-77	02.04.2019	wild boar	X		X
MWP2019-78	02.04.2019	pig	X		X
MWP2019-79	04.04.2019	wild boar	X		X
MWP2019-80	04.04.2019	pig	X		X
MWP2019-81	04.04.2019	wild boar	X		X
MWP2019-82	02.04.2019	pig	X		X
MWP2019-83	02.04.2019	pig	X		X
MWP2019-84	09.04.2019	pig	X		X
MWP2019-85	09.04.2019	pig	X		X
MWP2019-86	10.04.2019	pig	X		X
MWP2019-87	10.04.2019	pig	X		X
MWP2019-88	10.04.2019	pig	X		X
MWP2019-89	11.04.2019	pig	X		X
MWP2019-90	11.04.2019	pig	X		X
MWP2019-92	11.04.2019	pig	X		X
MWP2019-93	12.04.2019	pig	X		X
MWP2019-94	12.04.2019	pig	X		X
MWP2019-95	16.04.2019	pig	X		X
MWP2019-96	16.04.2019	pig	X		X
MWP2019-97	16.04.2019	pig	X		X
MWP2019-98	16.04.2019	pig	X		X
MWP2019-99	16.04.2019	pig	X		X
MWP2019-100	16.04.2019	pig	X		X
MWP2019-101	16.04.2019	pig	X		X
MWP2019-102	16.04.2019	pig	X		X
MWP2019-103	16.04.2019	pig	X		X
MWP2019-104	16.04.2019	pig	X		X
MWP2019-105	17.04.2019	pig	X		X

MWP2019-106	17.04.2019	pig	X		X
MWP2019-107	24.04.2019	pig	X		X
MWP2019-108	24.04.2019	pig	X		X
MWP2019-109	24.04.2019	pig	X		X
MWP2019-110	25.04.2019	pig	X		X
MWP2019-111	25.04.2019	pig	X		X
MWP2019-112	29.04.2019	pig	X		X
MWP2019-113	29.04.2019	pig	X		X
MWP2019-114	29.04.2019	pig	X		X
MWP2019-115	29.04.2019	pig	X		X
MWP2019-116	29.04.2019	pig	X		X
MWP2019-117	30.04.2019	wild boar	X		X
MWP2019-118	30.04.2019	pig	X		X
MWP2019-119	30.04.2019	pig	X		X
MWP2019-120	30.04.2019	pig	X		X
MWP2019-121	30.04.2019	pig	X		X
MWP2019-122	06.05.2019	wild boar	X		X
MWP2019-123	09.05.2019	pig	X		X
MWP2019-124	17.05.2019	pig	X		X
MWP2019-125	17.05.2019	pig	X		X
MWP2019-126	17.05.2019	pig	X		X
MWP2019-127	22.05.2019	pig	X		X
MWP2019-128	22.05.2019	pig	X		X
MWP2019-129	22.05.2019	pig	X		X
MWP2019-130	22.05.2019	pig	X		X
MWP2019-131	23.05.2019	pig	X		X
MWP2019-132	24.05.2019	wild boar	X		X
MWP2019-133	24.05.2019	wild boar	X		X
MWP2019-134	28.05.2019	pig	X		X
MWP2019-135	28.05.2019	pig	X		X
MWP2019-136	28.05.2019	pig	X		X
MWP2019-137	28.05.2019	pig	X		X
MWP2019-138	28.05.2019	pig	X		X
MWP2019-139	28.05.2019	pig	X		X
MWP2019-140	28.05.2019	pig	X		X
MWP2019-141	28.05.2019	pig	X		X
MWP2019-142	29.05.2019	pig	X		X
MWP2019-143	29.05.2019	pig	X		X
MWP2019-144	29.05.2019	pig	X		X
MWP2019-145	03.06.2019	pig	X		X
MWP2019-146	04.06.2019	pig	X		X
MWP2019-147	04.06.2019	pig	X		X
MWP2019-148	04.06.2019	pig	X		X
MWP2019-149	05.06.2019	pig	X		X
MWP2019-150	06.06.2019	pig	X		X
MWP2019-151	06.06.2019	pig	X		X
MWP2019-152	06.06.2019	pig	X		X
MWP2019-153	06.06.2019	pig	X		X
MWP2019-154	07.06.2019	pig	X		X
MWP2019-155	07.06.2019	pig	X		X
MWP2019-156	07.06.2019	pig	X		X
MWP2019-157	07.06.2019	pig	X		X

MWP2019-158	11.06.2019	pig	X		X
MWP2019-159	13.06.2019	pig	X		X
MWP2019-160	13.06.2019	pig	X		X
MWP2019-161	14.06.2019	pig	X		X
MWP2019-162	14.06.2019	wild boar	X		X
MWP2019-163	18.06.2019	pig	X		X
MWP2019-164	18.06.2019	pig	X		X
MWP2019-165	18.06.2019	pig	X		X
MWP2019-166	18.06.2019	pig	X		X
MWP2019-167	18.06.2019	pig	X		X
MWP2019-168	19.06.2019	pig	X		X
MWP2019-169	24.06.2019	pig	X		X
MWP2019-170	24.06.2019	pig	X		X
MWP2019-171	25.06.2019	wild boar	X		X
MWP2019-172	26.06.2019	pig	X		X
MWP2019-173	26.06.2019	pig	X		X
MWP2019-174	26.06.2019	pig	X		X
MWP2019-175	28.06.2019	pig	X		X
MWP2019-176	28.06.2019	pig	X		X
MWP2019-177	28.06.2019	pig	X		X
MWP2019-178	01.07.2019	wild boar	X		X
MWP2019-179	03.07.2019	pig	X		X
MWP2019-180	03.07.2019	pig	X		X
MWP2019-181	03.07.2019	pig	X		X
MWP2019-182	03.07.2019	pig	X		X
MWP2019-183	05.07.2019	wild boar	X		X
MWP2019-184	09.07.2019	pig	X		X
MWP2019-185	09.07.2019	pig	X		X
MWP2019-186	09.07.2019	pig	X		X
MWP2019-187	09.07.2019	pig	X		X
MWP2019-188	09.07.2019	pig	X		X
MWP2019-189	09.07.2019	pig	X		X
MWP2019-190	09.07.2019	pig	X		X
MWP2019-191	09.07.2019	pig	X		X
MWP2019-192	09.07.2019	pig	X		X
MWP2019-193	10.07.2019	pig	X		X
MWP2019-194	10.07.2019	pig	X		X
MWP2019-195	10.07.2019	pig	X		X
MWP2019-196	10.07.2019	pig	X		X
MWP2019-197	10.07.2019	pig	X		X
MWP2019-198	10.07.2019	pig	X		X
MWP2019-199	10.07.2019	pig	X		X
MWP2019-200	10.07.2019	pig	X		X
MWP2019-201	10.07.2019	pig	X		X
MWP2019-202	10.07.2019	pig	X		X
MWP2019-203	10.07.2019	pig	X		X
MWP2019-204	16.07.2019	pig	X		X
MWP2019-205	17.07.2019	pig	X		X
MWP2019-206	18.07.2019	pig	X		X
MWP2019-207	23.07.2019	pig	X		X
MWP2019-208	23.07.2019	pig	X		X
MWP2019-209	23.07.2019	pig	X		X

MWP2019-210	23.07.2019	pig	X		X
MWP2019-211	24.07.2019	pig	X	X	X
MWP2019-212	24.07.2019	pig	X	X	X
MWP2019-213	24.07.2019	pig	X	X	X
MWP2019-214	24.07.2019	pig	X	X	X
MWP2019-215	25.07.2019	pig	X	X	X
MWP2019-216	25.07.2019	pig	X	X	X
MWP2019-217	30.07.2019	pig	X	X	X
MWP2019-218	11.07.2019	pig	X		X
MWP2019-219	01.08.2019	pig	X	X	X
MWP2019-220	01.08.2019	wild boar	X	X	X
MWP2019-221	05.08.2019	wild boar	X	X	X
MWP2019-222	07.08.2019	pig	X	X	X
MWP2019-223	07.08.2019	pig	X	X	X
MWP2019-224	07.08.2019	pig	X	X	X
MWP2019-225	12.08.2019	wild boar	X	X	X
MWP2019-226	12.08.2019	wild boar	X	X	X
MWP2019-227	12.08.2019	wild boar	X	X	X
MWP2019-228	14.08.2019	pig	X	X	X
MWP2019-229	14.08.2019	pig	X	X	X
MWP2019-230	16.08.2019	pig	X	X	X
MWP2019-231	16.08.2019	wild boar	X	X	X
MWP2019-232	19.08.2019	pig	X	X	X
MWP2019-233	19.08.2019	pig	X	X	X
MWP2019-234	19.08.2019	pig	X	X	X
MWP2019-235	20.08.2019	wild boar	X	X	X
MWP2019-236	20.08.2019	wild boar	X	X	X
MWP2019-237	22.08.2019	pig	X	X	X
MWP2019-238	22.08.2019	wild boar	X	X	X
MWP2019-239	22.08.2019	wild boar	X	X	X
MWP2019-240	22.08.2019	wild boar	X	X	X
MWP2019-241	23.08.2019	wild boar	X	X	X
MWP2019-242	29.08.2019	pig	X	X	X
MWP2019-243	29.08.2019	wild boar	X	X	X
MWP2019-244	29.08.2019	pig	X	X	X
MWP2019-245	29.08.2019	pig	X	X	X
MWP2019-246	30.08.2019	pig	X	X	X
MWP2019-247	30.08.2019	pig	X	X	X
MWP2019-248	30.08.2019	wild boar	X	X	X
MWP2019-249	03.09.2019	wild boar	X	X	X
MWP2019-250	03.09.2019	wild boar	X	X	X
MWP2019-251	03.09.2019	wild boar	X	X	X
MWP2019-252	03.09.2019	wild boar	X	X	X
MWP2019-253	03.09.2019	pig	X	X	X
MWP2019-254	04.09.2019	pig	X	X	X
MWP2019-255	05.09.2019	pig	X	X	X
MWP2019-256	05.09.2019	pig	X	X	X
MWP2019-257	05.09.2019	pig	X	X	X
MWP2019-258	05.09.2019	wild boar	X	X	X
MWP2019-259	05.09.2019	pig	X	X	X
MWP2019-260	05.09.2019	pig	X	X	X
MWP2019-261	12.09.2019	pig	X	X	X

MWP2019-262	05.09.2019	wild boar	X	X	X
MWP2019-263	09.09.2019	wild boar	X	X	X
MWP2019-264	09.09.2019	wild boar	X	X	X
MWP2019-265	09.09.2019	wild boar	X	X	X
MWP2019-266	11.09.2019	wild boar	X	X	X
MWP2019-267	11.09.2019	wild boar	X	X	X
MWP2019-268	13.09.2019	wild boar	X	X	X
MWP2019-269	13.09.2019	wild boar	X	X	X
MWP2019-270	13.09.2019	pig	X	X	X
MWP2019-271	13.09.2019	pig	X	X	X
MWP2019-272	17.09.2019	pig	X	X	X
MWP2019-273	17.09.2019	pig	X	X	X
MWP2019-274	17.09.2019	pig	X	X	X
MWP2019-275	20.09.2019	pig	X	X	X
MWP2019-276	20.09.2019	pig	X	X	X
MWP2019-277	20.09.2019	pig	X	X	X
MWP2019-278	23.09.2019	wild boar	X		X
MWP2019-279	24.09.2019	pig	X	X	X
MWP2019-280	25.09.2019	wild boar	X	X	X
MWP2019-281	25.09.2019	pig	X	X	X
MWP2019-282	25.09.2019	pig	X	X	X
MWP2019-283	25.09.2019	pig	X	X	X
MWP2019-284	27.09.2019	pig	X	X	X
MWP2019-285	27.09.2019	pig	X	X	X
MWP2019-286	27.09.2019	pig	X	X	X
MWP2019-287	30.09.2019	wild boar	X	X	X
MWP2019-288	01.10.2019	pig	X	X	X
MWP2019-289	07.10.2019	pig	X	X	X
MWP2019-290	08.10.2019	pig	X	X	X
MWP2019-291	09.10.2019	wild boar			X
MWP2019-292	09.10.2019	wild boar	X	X	X
MWP2019-293	08.10.2019	pig	X	X	X
MWP2019-294	09.10.2019	pig	X	X	X
MWP2019-295	09.10.2019	pig	X	X	X
MWP2019-296	10.10.2019	pig	X		X
MWP2019-297	11.10.2019	wild boar	X	X	X
MWP2019-298	11.10.2019	pig	X	X	X
MWP2019-299	15.10.2019	pig	X	X	X
MWP2019-300	15.10.2019	pig	X	X	X
MWP2019-301	16.10.2019	pig	X	X	X
MWP2019-302	16.10.2019	pig	X	X	X
MWP2019-303	16.10.2019	pig	X	X	X
MWP2019-304	16.10.2019	pig	X	X	X
MWP2019-305	17.10.2019	wild boar	X	X	X
MWP2019-306	21.10.2019	pig	X	X	X
MWP2019-307	21.10.2019	pig	X	X	X
MWP2019-308	21.10.2019	pig	X	X	X
MWP2019-309	22.10.2019	pig	X	X	X
MWP2019-310	22.10.2019	pig	X	X	X
MWP2019-311	22.10.2019	pig	X	X	X
MWP2019-312	22.10.2019	wild boar		X	
MWP2019-313	24.10.2019	pig	X	X	X

MWP2019-314	24.10.2019	pig	X	X	X
MWP2019-315	24.10.2019	pig	X	X	X
MWP2019-316	24.10.2019	pig	X	X	X
MWP2019-317	24.10.2019	pig	X	X	X
MWP2019-318	25.10.2019	pig	X	X	X
MWP2019-319	25.10.2019	pig	X	X	X
MWP2019-320	25.10.2019	pig	X	X	X
MWP2019-321	28.10.2019	wild boar	X	X	X
MWP2019-322	28.10.2019	wild boar	X	X	X
MWP2019-323	29.10.2019	pig	X	X	X
MWP2019-324	30.10.2019	pig	X	X	X
MWP2019-325	30.10.2019	wild boar	X	X	X
MWP2019-326	30.10.2019	pig	X	X	X
MWP2019-327	30.10.2019	pig	X	X	X
MWP2019-328	30.10.2019	pig	X	X	X
MWP2019-329	04.11.2019	pig	X	X	X
MWP2019-330	04.11.2019	pig	X	X	X
MWP2019-331	04.11.2019	pig	X	X	X
MWP2019-332	04.11.2019	pig	X	X	X
MWP2019-333	04.11.2019	pig	X	X	X
MWP2019-334	04.11.2019	pig	X	X	X
MWP2019-335	04.11.2019	pig	X	X	X
MWP2019-336	04.11.2019	pig	X	X	X
MWP2019-337	04.11.2019	pig	X	X	X
MWP2019-338	04.11.2019	pig	X	X	X
MWP2019-339	05.11.2019	wild boar	X	X	X
MWP2019-340	05.11.2019	wild boar	X	X	X
MWP2019-341	05.11.2019	wild boar	X	X	X
MWP2019-342	05.11.2019	wild boar	X	X	X
MWP2019-343	05.11.2019	wild boar	X	X	X
MWP2019-344	06.11.2019	wild boar	X	X	X
MWP2019-345	11.11.2019	wild boar	X		X
MWP2019-346	12.11.2019	pig	X	X	X
MWP2019-347	12.11.2019	pig	X	X	X
MWP2019-348	12.11.2019	pig	X	X	X
MWP2019-349	12.11.2019	pig	X	X	X
MWP2019-350	12.11.2019	wild boar	X	X	X
MWP2019-351	15.11.2019	pig	X	X	X
MWP2019-352	15.11.2019	pig	X	X	X
MWP2019-353	18.11.2019	pig	X	X	X
MWP2019-354	18.11.2019	pig	X	X	X
MWP2019-355	18.11.2019	pig	X	X	X
MWP2019-356	18.11.2019	pig	X	X	X
MWP2019-357	18.11.2019	pig	X	X	X
MWP2019-358	18.11.2019	pig	X	X	X
MWP2019-359	18.11.2019	pig	X	X	X
MWP2019-360	21.11.2019	pig	X	X	X
MWP2019-361	26.11.2019	wild boar	X	X	X
MWP2019-362	27.11.2019	pig	X	X	X
MWP2019-363	27.11.2019	wild boar	X	X	X
MWP2019-364	28.11.2019	pig	X	X	X
MWP2019-365	28.11.2019	pig	X	X	X

MWP2019-366	19.11.2019	pig	X	X	X
MWP2019-367	19.11.2019	wild boar	X	X	X
MWP2019-368	27.11.2019	wild boar	X	X	X
MWP2019-369	28.11.2019	pig	X	X	X
MWP2019-370	29.11.2019	pig	X	X	X
MWP2019-371	02.12.2019	pig	X	X	X
MWP2019-372	02.12.2019	pig	X	X	X
MWP2019-373	02.12.2019	pig	X	X	X
MWP2019-374	03.12.2019	wild boar	X	X	X
MWP2019-375	05.12.2019	pig	X	X	X
MWP2019-376	06.12.2019	pig	X	X	X
MWP2019-377	09.12.2019	pig	X	X	X
MWP2019-378	10.12.2019	pig	X	X	X
MWP2019-379	10.12.2019	pig	X	X	X
MWP2019-380	10.12.2019	pig	X	X	X
MWP2019-381	10.12.2019	pig	X	X	X
MWP2019-382	11.12.2019	wild boar	X	X	X
MWP2019-383	13.12.2019	wild boar	X	X	X
MWP2019-384	17.12.2019	pig	X	X	X
MWP2019-385	17.12.2019	wild boar	X	X	X
MWP2019-386	17.12.2019	wild boar	X	X	X
MWP2019-387	18.12.2019	pig	X	X	X
MWP2019-388	18.12.2019	pig	X	X	X
MWP2019-389	18.12.2019	pig	X	X	X
MWP2019-390	18.12.2019	pig	X	X	X
MWP2019-391	19.12.2019	pig	X	X	X
MWP2019-392	19.12.2019	pig	X	X	X
MWP2019-393	23.12.2019	wild boar	X	X	X
MWP2019-394	23.12.2019	pig	X	X	X
MWP2019-395	30.12.2019	wild boar	X	X	X

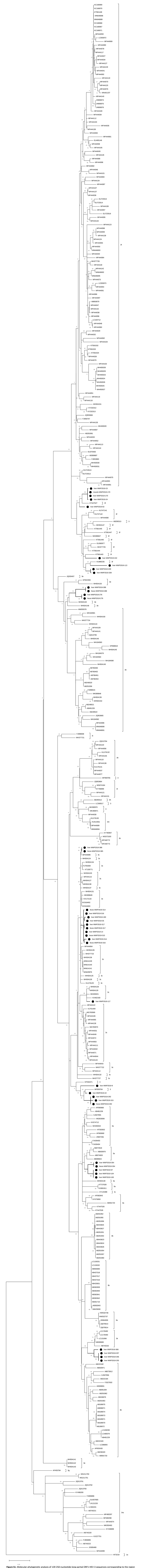

Figure S1. Molecular phylogenetic analysis of 139 250-nucleotide-long partial ORF1 HEV-3 sequences corresponding to the region 127–376 of reference sequence FJ705359.
